# Supplementary material for: Consumers’ risk perception, market demand, and firm innovation: Evidence from China
Source: PLoS One. 2024 May 17;19(5):e0301802. doi: 10.1371/journal.pone.0301802 (PMC11101097; doi:10.1371/journal.pone.0301802)
Supplement: S1 Table — (DOCX) [file pone.0301802.s001.docx]

**Table S1 Category of the treatment group**

| **Category** | **Industry code** | **Instruction** |
| --- | --- | --- |
| Daily chemical products | 2671 | Soap and synthetic detergent manufacturing |
|  | 2672 | Cosmetics Manufacturing |
|  | 2673 | Oral cleaning products manufacturing |
|  | 2674 | Spices, essence manufacturing |
|  | 2679 | Other daily chemical products manufacturing |
| Pharmaceutical Manufacturing | 2710 | Chemical original drug manufacturing |
|  | 2720 | Chemical pharmaceutical preparation manufacturing |
|  | 2730 | Chinese patent medicine manufacturing |
|  | 2740 | Veterinary medicine manufacturing |
|  | 2750 | Veterinary medicine manufacturing |
|  | 2760 | Biological, biochemical products manufacturing |
|  | 2770 | Health materials and medical supplies manufacturing |
