# Supplementary material for: Consumers’ risk perception, market demand, and firm innovation: Evidence from China
Source: PLoS One. 2024 May 17;19(5):e0301802. doi: 10.1371/journal.pone.0301802 (PMC11101097; doi:10.1371/journal.pone.0301802)
Supplement: S2 Table — (DOCX) [file pone.0301802.s002.docx]

**Table S2 Daily chemical products produced by pharmaceutical firms**

| **Firm** | **Represent products or categories** |
| --- | --- |
| Yunnan Baiyao | Toothpaste |
| Panlong Yunhai | Cosmeceutical |
| Mayinglong | Eye cream |
| Tongrentang | Medica |
| Sanjing Pharmaceutical | Toothpaste |
| Renhe Pharmaceutical | Toothpaste, hair dye |
| Pien Tze Huang | Cosmeceutical |
| Conn Beffri | Maternity care products |
| Worship Hall | Osmeceutical |
| Wanglaoji | Acne gel |
| isheng Pharmaceutical | Cosmeceutical |
| Hua Shen Group | Cosmeceutical |
| Dihong Pharmaceutical | Soap, toothpaste |
| Dong-e-e-jiao | Facial mask |
| Huabei Pharmaceutical | Cosmeceutical |
| Revision Pharmaceutical Group | Cosmeceutical |
| Jiangzhong Group | Steam eye mask |
| Harbin Medicine Group | Cosmeceuticals |
| Wuhan Jianmin | Baby care products |
