# Supplementary material for: Consumers’ risk perception, market demand, and firm innovation: Evidence from China
Source: PLoS One. 2024 May 17;19(5):e0301802. doi: 10.1371/journal.pone.0301802 (PMC11101097; doi:10.1371/journal.pone.0301802)
Supplement: S1 Appendix — (DOCX) [file pone.0301802.s003.docx]

# S1 Appendix

**Calculating Similarity Between Industrial Sectors**

The “indirect R&D investment” received by industry $j$ due to inter-industry technology spillover can be defined as:

$IRD_{j}=\sum_{i\neq j} w_{ij}RD_{i}$ (B.1)

where $RD_{i}$represents the direct R&D investment of industry $i$, and $w_{ij}$ represents the weight of the technology acquired by industry $j$ in the total technology stock of industry $i$ through the spillover effect.

Equation (B.1) uses the direct consumption coefficient in the input-output table as the weight to calculate the indirect R&D investment between different industries [78,79]. The similarity between industrial sectors is defined as:

$w_{ij}=\frac{\sum_{k} a_{ki}a_{kj}}{\sqrt{\sum_{k} a_{ki}^{2}\cdot\sum_{k} a_{kj}^{2}}}$ (B.2)

In Equation (B.2), $a_{ki}$ and$a_{kj}$ are the $i$th and $j$th industries, respectively, to obtain the element at the $k$th position of the column vector of the direct consumption coefficient matrix. The more similar the two industries are, the closer the Angle cosine is to 1.

This paper draws on this method and uses the daily chemical industry as a reference to calculate the similarity between other industries and the daily chemical industry, then calculates the indirect R&D obtained by the daily chemical industry from other industries through inter-industry spillover. The daily chemical industry is taken as the reference industry, and the similarity between other industries and the daily chemical industry is calculated according to Equation (2B).

This paper first calculates the similarity $w_{ij}$ among various industrial sectors according to the direct consumption coefficient matrix of input-output tables in 2007, 2012, and 2017. As the similarity will decrease with the increase of year span, the simple weighted average method is chosen to calculate the similarity between 2008 and 2011 and 2013 and 2014:

$w_{ij,t}=\left( 1-\frac{t-2007}{5} \right)w_{ij,2007}+\left( \frac{t-2007}{5} \right)w_{ij,2012}$, *t*=2008,2009,2010,2011 (B.3)

$w_{ij,t}=\left( 1-\frac{t-2012}{5} \right)w_{ij,2012}+\left( \frac{t-2012}{5} \right)w_{ij,2017},$*t*=2013,2014 (B.4)

where $w_{ijt}$is indirect R&D investment spillover from industry j to industry i, in which the internal expenditure data of scientific and technological activities of firms above the designated size in each industry measure R&D investment.
