# Supplementary material for: Consumers’ risk perception, market demand, and firm innovation: Evidence from China
Source: PLoS One. 2024 May 17;19(5):e0301802. doi: 10.1371/journal.pone.0301802 (PMC11101097; doi:10.1371/journal.pone.0301802)
Supplement: Attachment — Submitted filename: Response to reviewer1.docx [file pone.0301802.s005.docx]

**Response to Reviewer #1:**

This research article explores the relationship between consumers' risk perception and firm innovation, using the Bawang Shampoo Causes Cancer Event as a case study. While the study provides some valuable insights, there are several aspects to consider such as:

**Reply:** We thank the anonymous reviewer very much for reading the manuscript so carefully, and offering so many important and constructive comments. We have benefited tremendously from your constructive comments and suggestions, which are all extremely helpful to bring the paper up to the standard. We have tried to make changes and revisions to make the research theme more prominent and the logic clearer. Please allow us to take this opportunity to send our greatest appreciation to you. We try to address these problems and the flaws you point out in the following.

**Question 1:** The paper inconsistently uses different citation styles (e.g., some references are in APA style, while others follow an alternative style). A consistent and proper citation style should be used throughout the paper.

**Reply:** We sincerely thank the reviewer for careful reading. We have meticulously revised the citation style and ensured consistent citation style throughout the manuscript. We believe these changes contribute to the overall clarity and professionalism of the document.

**Question 2:** The conclusion provides a brief summary of the research, but it could be more focused on summarizing the key findings and their implications. It would also be beneficial to highlight limitations and suggest directions for future research.
 **Reply:** Thanks for this valuable comment. We have revised this part according to your suggestion (pp.19-22).

(i) We have now summarized our research content and methods, and explained our main empirical findings and their implications in the conclusion part.

“ In this paper, we examine the links between consumers’ risk perception, market demand, and firm innovation, taking advantage of the disclosure and the extensive reporting of the Bawang event in 2010. The theoretical hypothesis section of this paper argues that when a sudden accident occurs, such as a product harm crisis, consumers’ risk perception tends to increase, which, in turn, affects their purchasing behavior. This change in consumer behavior leads to a shift in market demand, eventually influencing manufacturing enterprises’ innovation activities. This paper employs a difference-in-differences approach by using the Bawang event as a quasi-natural experiment to identify the impact of consumers’ risk perception changes on firm innovation. The findings are as follows.

First, consumers’ risk perception effectively promotes firms to innovate. The increase in consumers’ risk perception of products containing dioxane after the Bawang event led to a significant increase in patent applications in the daily chemical and pharmaceutical manufacturing sectors, which rose by approximately 7.6% compared to other manufacturing industries. We propose that consumers’ risk perception affects firm innovation by influencing market demand and provide direct evidence to support this mechanism. In a sudden accident, consumer demand can decrease, leading to a firm’s profits and market share decline. Firms need to take appropriate measures to regain consumer trust and reduce their perception of risk. However, changing factor input or withdrawing from the market can be expensive for firms. Thus, implementing technological or product innovation is their best choice.

Second, we examine the impact of the position in the industrial chain and technology spillover on consumers’ perceived risk and firm innovation. Our study has revealed two findings: First, within the same industry chain, the downstream industry is more vulnerable to changes in consumer demand due to its closer proximity to consumers and, therefore, more sensitive to risk perception. This sensitivity makes changes in innovation activities more noticeable in the downstream industry. Second, cross-industry technology spillovers are more likely to occur between firms and industries with high technical similarity when a sudden accident occurs. The higher the technological similarity with the firm involved, the more significant the impact of consumers’ risk perception on the innovation of the enterprise.”

(ii) We have discussed the contributions and limitations of our research, and future research directions (pp.20-22).

Compared with the existing research, we have provided the possible marginal contribution of our manuscript (1) The first is the research perspective. While there is ample literature on how market demand drives innovation, there is little research on how consumers’ risk perception affects demand-driven innovation. Our research aims to fill this gap and provide a comprehensive understanding of the subject. In addition, our research also examines the strategies that firms can adopt to restore consumer trust and reduce risk perception following product harm crises. While previous studies have mainly focused on external strategies, such as crisis PR and advertising, we find that internal strategies, such as improving product quality and technology through innovation, can also play a crucial role in restoring consumer trust and reducing perceived risks. (2) The second is the research methods. Previous studies on "demand-driven innovation" have not been successful in solving the endogenous problem and obtaining an unbiased and consistent estimate. However, this paper addresses this issue by selecting the Bawang event as an exogenous shock and using the DID approach method to avoid endogeneity problems. (3) The third is the theoretical mechanisms. This paper introduces economic concepts such as industrial chain, industrial correlation, and technology spillover effect in the research framework on the impact of consumers’ risk perception on firm innovation. It is the first study that finds a relationship between the influence of consumers’ risk perception on firm innovation and their position in the industrial chain and technical similarity.

We have also discussed the limitations of our research and the future research directions. During our discussion, we explored the extent to which the results we obtained can be applied or generalized to other contexts. While our empirical findings may have limited applicability, the research concepts and theoretical framework can still be used to investigate similar issues. For instance, we can apply them to study the impact of changes in consumers' perceived risks on market demand and innovation during unexpected events like the COVID-19 pandemic (pp.21-22). In addition, this paper specifically analyzes the changes in risk perception from the perspectives of social risk and functional risk, and it is more detailed than previous studies. However, like previous studies, the discussion on consumers’ risk perception in this paper is still a qualitative analysis. In the future, constructing quantitative indicators of consumers’ risk perception into the empirical model will become a focus of subsequent research.

**Question 3:** The article briefly mentions policy implications but could provide a more detailed discussion of how the findings could inform business strategies, government policies, and consumer behavior. Practical recommendations based on the research findings would enhance the article's relevance.
 **Reply:** Thank you for this suggestion. We have revised this section to elaborate on the policy implications based on our findings (pp.20-21).

“The research content and empirical results of this paper have policy implications for the real world today, mainly in three ways.

(1) For the government. This paper examines how consumer risk perception affects firm innovation and how the industrial chain location and technological similarity influence it. The study suggests that when the government formulates industrial and innovation policies, it needs to consider the role of the industrial chain and the spillover effect. The government should create matching policy programs for different types of firms. On the one hand, the government should prioritize the development of leading and high-impact industries and increase investment and policy support for them. This approach would promote the common development of other sectors. On the other hand, the government should recognize that some firms may have weak innovation ability and enthusiasm and may struggle to cope with demand-side shocks. To encourage them, the government can stimulate the internal motivation of these firms to innovate through tax incentives, subsidies, and other policies. Additionally, the government should improve the merger and reorganization mechanism of firms. It should eliminate firms with low technical levels and optimize industries through resource integration and factor reconstruction.

(2) For the firm. Firms should take advantage of the spillover effect of innovation and participate actively in collaborative innovation activities within their supply chain while optimizing their innovation strategies. Specifically, firms should closely monitor the innovation dynamics of related companies and learn from and absorb their innovation results to improve their product quality and production efficiency, thereby enhancing their competitiveness. Additionally, firms should maintain timely and effective interaction with industry-related businesses to keep track of their innovation plans and the development progress of new products. By collaborating with other firms and sharing resources and knowledge, firms can improve their innovation ability and performance. For instance, they can jointly establish joint research and development teams with other firms to develop new technologies and products, reducing costs, shortening research and development cycles, and improving market competitiveness.

(3) For the consumers. This paper's research results indicate that consumers' perceived risk can influence market demand, affecting firms' innovation. Therefore, both governments and firms need to pay heed to the consumers. Amidst the intensifying downward risks of the global economy, the government should focus on promoting household consumption and expanding domestic demand. To achieve this, the government can stimulate the willingness of consumers to consume by providing them with cash subsidies, consumer vouchers, and other incentives. When the market demand is strong, there are sufficient incentives for firms to invest in research and development projects, improve production efficiency, and increase product diversity, thus promoting economic growth. Firms should listen to consumers' opinions and feedback during production, operation, and research and development and amend their business strategy and research and development direction accordingly. On the other hand, consumers should actively exercise and maintain their right to supervision, right to know, and other relevant rights and collaborate with the government to supervise and give feedback on relevant firms and products. This would encourage firms to provide better products and services to the consumers.”

**Question 4:** The paper proposes a theoretical framework but does not deeply explain the theoretical underpinnings of the study, especially in the context of consumer behavior and innovation. It would be helpful to discuss how consumer risk perception aligns with established theories in economics and consumer psychology. The choice of the "difference-in-differences" (DID) approach is suitable.
 **Reply:** Thanks for this helpful comment. We have revised Section 2 (pp.3-7) of our research paper according to the reviewer's suggestion.

Our theoretical framework is based on the idea that when a sudden accident occurs, such as a product harm crisis, consumers’ risk perception tends to increase, which, in turn, affects their purchasing behaviour. This change in consumer behaviour leads to a shift in market demand, eventually influencing manufacturing enterprises’ innovation activities. Our theoretical underpinnings are developed based on this logic.

First, we have reviewed relevant literature on consumer psychology to explore how sudden accidents impact consumers' perceived risk, affect their behavior, and ultimately influence market demand. Based on our findings, we have proposed a research hypothesis:

Hypothesis 1 (H1). Sudden accident has an impact on consumers’ risk perception.

Hypothesis 2 (H2). Consumers’ risk perception has an impact on market demand.

Then, we have delved into the relevant economics literature to explore how demand influences innovation. We will then present our research hypothesis:

Hypothesis 3 (H3). Market demand has an impact on firm innovation.

Finally, we have examined how consumers' risk perception drives innovation as a demand-pulling force, highlighting its differences from other demand-pulling forces. Furthermore, we have introduced economic concepts such as industrial chain, industrial linkage, and technology spillover into the research of "demand-driven innovation" under the framework of risk perception.

Hypothesis 4 (H4). Consumers’ risk perception has an impact on firm innovation.

Hypothesis 5 (H5). Consumers’ risk perception has an impact on firm innovation, and the extent of the impact depends on the firm’s position in the industrial chain.

Hypothesis 6 (H6). Consumers’ risk perception has an impact on firm innovation and the extent of the impact related to technology spillover.

Through the discussion of these three topics, we have connected consumers’ risk perception with the literature on consumer psychology and economics. Our research has covered several topics, and to establish a better connection between them, we have divided Section 2 into three parts: "Consumers' Risk Perception and Market Demand", "Market Demand and Firm Innovation", and "Consumers' Risk Perception and Firm Innovation". In each section, we have explained our theoretical underpinnings and relevant concepts, reviewed and commented on research about relevant topics, and presented our research hypotheses and theoretical frameworks. We have not presented the full contents of the revisions here. However, they can be found in the revised paper (pp.3-7).

We chose the DID approach as our research method for the following reasons. Previous studies on "demand-driven innovation" have not been successful in solving the endogenous problem and obtaining an unbiased and consistent estimate. Our paper addresses this issue by selecting the Bawang event as an exogenous shock and using the DID approach method to avoid endogeneity problems. The accidental nature of the Bawang event and the extensive media coverage at the time provide quite convincing evidence of the externality of our shock.

**Question 5:** There are minor language issues, such as grammatical errors and awkward sentence structures, that could be addressed for improved clarity and readability.

**Reply:** We apologize for the language issue of our manuscript. We tried our best to improve the manuscript and made some changes to the manuscript. We did not list the changes here but marked them in red in the revised paper. We appreciate for reviewer's warm work earnestly and hope that the correction will meet with approval.

**Question 6:** The paper focuses on a specific event, the Bawang Shampoo Causes Cancer Event in China. This limited scope might hinder the generalizability of the findings to other contexts. The paper should discuss the limitations and potential implications for the applicability of the results to other regions or industries.

**Reply:** Thank you for this suggestion. We have added a paragraph in the conclusion to discuss our research's limitations and the possible directions for future research (pp.21-22).

“The paper primarily concentrates on the Bawang event and two specific industries, namely the pharmaceutical industry and the daily chemical industry. The empirical results obtained have a limited application scope. Nevertheless, the research ideas and theoretical framework can be applied to similar issues, such as the impact of changes in consumers' perceived risks on market demand and innovation when other unexpected events occur. Take COVID-19, for example. The outbreak of COVID-19 in early 2020 has had a significant impact on countries worldwide due to its rapid and widespread transmission and the difficulties in preventing and controlling it. Consumer demand has changed after the outbreak of COVID-19 due to increased risk perception. Residents have increased their spending on medical supplies to prevent the epidemic, leading to an increase in demand for products and services in the medical and healthcare fields. Additionally, residents have minimized going out and opted to isolate at home to prevent the spread of the virus, thus shifting their demand from offline to online platforms such as online retail, fresh food e-commerce, express logistics, online entertainment, online education, and remote work. The changes in consumers' risk perception and behaviour have forced relevant industries to carry out technological innovations to cope with changes in people's needs after the epidemic. The COVID-19 pandemic has impacted various industries and regions. In the future, researchers can utilize it as an external shock to analyze the effect of shifts in consumers' perceived risks on market demand and innovation during unexpected events. These findings will have broader applicability once data becomes available.”

Finally, we hope that the changes we have made solve all your concerns about the article. We are more than happy to make any further changes that will improve the paper. Thank you again for your time and help.
